# New Andean plump toad of the genus *Osornophryne* (Anura: Bufonidae) from Cerro Candelaria, Ecuador

**DOI:** 10.7717/peerj.19760

**Published:** 2025-07-23

**Authors:** Juan P. Reyes-Puig, Miguel A. Urgiles-Merchán, H. Mauricio Ortega-Andrade, Diego F. Cisneros-Heredia, Julio C. Carrión-Olmedo, Mario H. Yáñez-Muñoz

**Affiliations:** 1 Fundación EcoMinga Red de Protección de Bosques Amenazados, Quito, Pichincha, Ecuador; 2 Unidad de Investigación, Instituto Nacional de Biodiversidad (INABIO), Quito, Pichincha, Ecuador; 3 Fundación Oscar Efrén Reyes, Baños, Tungurahua, Ecuador; 4 Integrative Biology Laboratory, Universidad Regional Amazónica Ikiam, Tena, Napo, Ecuador; 5 Biogeography and Spatial Ecology Research Group, Life Sciences Faculty, Universidad Regional Amazónica Ikiam, Tena, Napo, Ecuador; 6 Laboratorio de Zoología Terrestre & Museo de Zoología, Instituto de Biodiversidad Tropical IBIOTROP, Colegio de Ciencias Biológicas y Ambientales, Universidad San Francisco de Quito USFQ, Quito, Pichincha, Ecuador; 7 Reserva: The Youth Land Trust, Washington, DC, United States of America

**Keywords:** Amphibia, Upper Pastaza watershed, Phylogenetics, *Osornophryne backshalli* sp. nov., *Osornophryne sumacoensis*, *Osornophryne simpsoni*, Eastern montane Andes, Speciation

## Abstract

The amphibian genus *Osornophryne* is endemic to the northern Andes of South America and has long been considered rare. Recent explorations in the humid montane forests of the upper Pastaza Valley have uncovered previously unknown species. Here, we describe a new Andean toad species from the central Ecuadorian Andes, identified through genetic analyses and distinctive morphological and cranial traits. *Osornophryne backshalli* sp. nov., from Cerro Candelaria in the upper Pastaza River basin, is closely related to *O. sumacoensis* from Sumaco Volcano. This new species is characterized by a uniquely short fifth toe relative to toes I–III, triangular papillae on the snout tip, an occipital fold, large subconical and conical warts on the body, dorsal surfaces Brownish Olive with Spectrum Yellow and Light Neutral Gray flecks, ventral surfaces Brownish Olive with Spectrum Yellow bright blotches. Our phylogenetic analyses revise the genus taxonomy by delimiting two well-supported clades: the *Osornophryne bufoniformis* species group and the *Osornophryne guacamayo* species group. Furthermore, we show that the Pastaza River does not constitute a geographical barrier for *Osornophryne* distribution. These findings emphasize the value of continued exploration to expand our understanding of this genus in the humid montane forests of the Ecuadorian Andes.

## Introduction

Bufonidae is a diverse family of anuran amphibians comprising 80 genera and around 750 species distributed nearly worldwide, constituting one of the largest family of amphibians, a great diversity and endemism in this family are concentrated in the tropical Andes (Frost, 2024).

Within Bufonidae, the genus *Osornophryne* (Ruiz-Carranza & Hernández-Camacho, 1976) is restricted to the northern tropical Andes of South America. This genus includes eleven small toad species inhabiting high elevations between 2000 and 4000 m in Colombia and Ecuador, spanning from the northern Cordillera Central of Colombia to the central Cordillera Real of Ecuador (Frost, 2024; Páez-Moscoso & Guayasamin, 2012; Yánez-Muñoz et al., 2010). Unlike other widespread South American bufonids such as *Rhaebo* and *Rhinella*, *Osornophryne* species have a limited and endemic distribution in the Andean region.

Species richness within *Osornophryne* is highest in Ecuador’s eastern Andes along the Cordillera Real, where approximately one third of the species have been described only in recent decades (Gluesenkamp & Guayasamin, 2008; Mueses-Cisneros, Yánez-Muñoz & Guayasamin, 2010; Cisneros-Heredia & Gluesenkamp, 2010; Páez-Moscoso, Guayasamin & Yánez-Muñoz, 2011).

Recent herpetological surveys conducted by Fundación Ecominga and INABIO over the past 15 years in the upper Rio Pastaza basin have revealed several new species with restricted distributions (Reyes-Puig et al., 2019; INABIO-Instituto Nacional de Biodiversidad et al., 2023). Cerro Candelaria, located south of the Rio Pastaza, stands out for its exceptional diversity and concentration of endemic anurans (Reyes-Puig et al., 2010; Reyes-Puig et al., 2014; Reyes-Puig et al., 2023; Reyes-Puig et al., 2024).

Field expeditions to this area have yielded new specimens of *Osornophryne* toads. Comprehensive genetic, morphological, and osteological analyses have facilitated the identification of a new species from Cerro Candelaria, which is described in this study. This work aims to expand the taxonomic understanding of *Osornophryne* by describing this new species and clarifying its phylogenetic relationships within the genus, thereby contributing to the knowledge of amphibian diversity in the upper Rio Pastaza watershed of the eastern Ecuadorian Andes.

## Materials & Methods

**Ethics statement.** To follow Ecuadorian laws, we use research permits to access genetic resources: N° MAE-DNB-CM-2019-0120, MAATE-ARSFC-2023-3346, MAATE-ARSFC-2024-0847 awarded by the Ministry of Environment, Water, and Ecological Transition of Ecuador. We observe the guidelines for the use of live amphibians and reptiles in field research, in agreement with Beaupre et al. (2004).

**Nomenclature and Taxonomy.** For species level recognition, we followed the species concept proposed by De Queiroz (2007), treating as new species those independent lineages of metapopulations that are evolving separately with several supporting lines of evidence.

Species description follows the last *Osornophryne* taxonomical revisions (Páez-Moscoso, Guayasamin & Yánez-Muñoz, 2011; Páez-Moscoso & Guayasamin, 2012). The electronic version of this article in Portable Document Format (PDF) constitutes a published work according to the International Code of Zoological Nomenclature (ICZN). Therefore, the new names contained in the electronic version are considered effectively published under the Code from the electronic edition alone. This published work and the nomenclatural acts it contains have been registered with ZooBank, the official online registry of the ICZN. The ZooBank Life Science Identifiers (LSIDs) can be accessed, and the associated information viewed, through any standard web browser by appending the LSID to the prefix http://zoobank.org/. The LSID for this publication is: urn:lsid:zoobank.org:pub:459B3032-0A5E-4BDA-8457-9D4D53885725. The online version of this work is archived and available from the following digital repositories: PeerJ, PubMed Central, SCIE, and CLOCKSS.

**Taxon sampling & specimen management.** In the course of botanical research carried out in 2019, the first individual of a new *Osornophryne* was discovered fortuitously. This finding led to the initiation of systematic monitoring of amphibians on Cerro Candelaria, which culminated in the collection of several new specimens of *Osonophryne*. Collection of individuals follows a standard and standardized techniques and methods of management for inventory and amphibian monitoring, proposed by Lips et al. (2001), and described in previous studies made by our team in the area (Yánez Muñoz, Reyes Puig & Morales Mite, 2013; INABIO-Instituto Nacional de Biodiversidad, 2023; Reyes-Puig et al., 2024).

During field expeditions, each captured specimen was assigned a unique number and was taken to the base camp in an individual plastic bag to confirm its sex and relative age (Angulo et al., 2006). We recorded the time and date of capture, type of vegetation, substrate, activity, and climatic conditions. To facilitate the recognition of dorsal, ventral, and flanks patterns, the specimens were photographed with a unique code number and stored in a catalog of photographic references.

Specimens were sacrificed following the preservation guidelines suggested by Rueda, Castro & Cortez (2006). A local anesthetic solution, specifically 2% lidocaine (prepared from its commercial formulation, Roxicaine), was used for euthanasia. Tissue and liver samples were collected for DNA extraction. Subsequently, the specimens were fixed in 10% formalin for 24 h and then preserved in 70% ethanol. Sex and age of individuals were determined by identification of secondary sexual characters (*i.e.,* nuptial pads, males with vocal clefts, and body size) and also by direct inspection of the gonads through dorsolateral incisions.

Adult females of *Osornophryne sumacoensis* (MZUTI 5147) and the new species (DHMECN 18363) were dissected, removing skin and muscles from the head and mandibles, to expose the shape and structure of the squamosal and prootic, with emphasis on the crista parotica (there are small variation in the shape and articulation with other skull bones between species). Additionally, dermestid beetles were used for two days until the bones were free of muscle tissue and then degreased with sodium dodecyl sulfate for 24 h, as described by Yánez-Muñoz et al. (2018) and Reyes-Puig et al. (2023).

**Morphology.** Examined specimens were sourced from regional and national herpetology repositories: Colección de Herpetología del Instituto Nacional de Biodiversidad (DHMECN). Quito, Ecuador; Instituto de Ciencias Naturales (ICN) Bogotá Colombia, and Universidad Tecnológica Equinoccial (MZUTI), Quito, Ecuador. Museum acronyms follow Frost (2024).

Examined specimens are listed in Appendix S1. Characters and systematics follow those used by Páez-Moscoso & Guayasamin (2012), and previous *Osornophryne* descriptions (Yánez-Muñoz et al., 2010; Páez-Moscoso, Guayasamin & Yánez-Muñoz, 2011). Study of the detailed morphology of the skull, crista parotica, and squamosal bone follows terminology used by Ruiz-Carranza & Hernández-Camacho (1976) Duellman & Trueb (1994), Vélez-Rodriguez (2005) Pramuk (2006), and Páez-Moscoso, Guayasamin & Yánez-Muñoz (2011). Additionally, we recorded differences between the structure and composition of palmar and plantar tubercles, across species, ranging from smooth and low flat tubercles to numerous rounded and elevated tubercles with callosities. Preserved specimens in the DHMECN collection were photographed in 70% ethanol with a digital Canon camera.

Morphometric characters follow Páez-Moscoso, Guayasamin & Yánez-Muñoz (2011). Measurements were taken with digital calipers to the nearest 0.01 mm, and are as follow: (1) snout–vent length (SVL = distance from tip of snout (excluding the proboscis) to posterior margin of vent); (2) tibia length (TIB = length of flexed hind leg from knee to heel); (3) foot length (FL = distance from base of inner metatarsal tubercle to tip of Toe IV); (4) hand lenght (Hal = distance from the base of the hand measured from the posterior border of the outer metatatarsal tubercle to the tip of finger III); (5) head length (HL = distance from tip of snout to articulation of jaw); (6) head width (HW = greatest width of head measured between jaw articulations); (7) interorbital distance (IOD = shortest distance between medial margins of upper eyelids); (8) upper eyelid width (EW = greatest width of eyelid measured perpendicular to medial axis of skull); (9) internarinal distance (IND = distance between internal borders of nos- trils); (10) eye–nostril distance (EN = distance from anterior corner of eye to posterior border of nostril); (11) snout–eye distance (SE = distance from anterior corner of the eye to the tip of the rostrum); (12) eye diameter (ED = distance between anterior and posterior corners of eye); (13) finger-III length (FIIIL = distance from proximal border of finger I to distal end of finger III); (14) finger-IV length (distance from proximal border of Finger I to distal end of Finger IV); (15) Toe-IV length (TIVL = distance from proximal edge of Toe I to distal tip of Toe IV); (16) Toe-V length (TVL = distance from proximal border edge of Toe I to distal tip of Toe V). Sexual maturity was determined by the presence of nuptial pads in adult males and convoluted oviducts in adult females. Color nomenclature follows Köhler (2012). Basic measurements of the skull include width and length of the skull, width of the brain case, width of the parasphenoid alae and length of the cultriform process.

**DNA extraction, amplification, and sequencing.** We worked in two different laboratories using Oxford Nanopore Technologies. At the Laboratorio de Secuenciamiento de Ácidos Nucleicos INABIO, we processed 21 samples.

At the Integrative Biology Laboratory, Universidad Regional Amazónica IKIAM, total DNA was extracted from liver tissue using the UltraClean^®^ Tissue & Cells DNA Isolation kit (MO-BIO Laboratories, Inc., Carlsbad, CA, USA), following the manufacturer’s instructions. We amplified a standard DNA barcode fragment of the mitochondrial 16S rRNA gene (primers 16sSar-L-F: CGCCTGTTTATCAAAAACAT; 16sSbr-H-R: CCGGTCTGAACTCAGATCACGT) *via* Polymerase chain reaction (PCR). Thermocycling conditions followed Pinto-Sánchez et al. (2012). PCR products were purified with an Exo I/SAP enzymatic clean-up and sequenced in Macrogen C*O.* Ltd. (South Korea).

To reduce PCR amplification and sequencing costs for longer mitochondrial regions, we implemented a different strategy at the INABIO.

At INABIO, DNA was extracted from liver tissue using GeneJET Genomic DNA purification Kit (K0722), following the manufacturer’s instructions. Instead of targeting short barcode regions, we amplified longer mitochondrial fragments (≥2,000 bp) encompassing the 12S rRNA and 16S rRNA. These longer amplicons were sequenced using Oxford Nanopore Technologies. PCR amplification, sequencing, and downstream bioinformatics followed the protocol described in Yánez-Muñoz et al. (2025). A character matrix was built with the new sequences (Table S1) and aligned using MAFFT v7.017 with default settings (Katoh & Standley, 2013). To optimize the sampling of *Osornophryne*, similar sequences were searched with BLAST for 16S in the GenBank database. Unaligned regions were visually edited and unambiguous regions were manually corrected.

### Phylogenetic analysis

We partitioned our matrix in 12S rRNA, tRNA-Val, and 16S rRNA genes to estimate the best evolutionary schemes for each partition. Substitution models and maximum likelihood tree inference were performed using IQTree (Trifinopoulos et al., 2016).

We ran a phylogenetic inference tree with 1,000 UFBoot (Ultra-Fast Bootstrap approximation approach), 1,000 maximum iterations, 0.99 minimum correlation coefficient, 1,000 replicates for SH-aLRT (Shimodaira–Hasegawa Approximate Likelihood Ratio Test) branch test and an approximate Bayes test (Guindon et al., 2010). Support values mentioned herein follows this format: SH-aLRT support (%)/ultrafast bootstrap support (%).

The percentage of bases/residues that are identical between sequences (% identity) was calculated on a 850 bp trimmed matrix of 16S rRNA to estimate p-genetic distances in MEGA 11 (Tamura, Stecher & Kumar, 2021).

## Results

**Figure 1:**
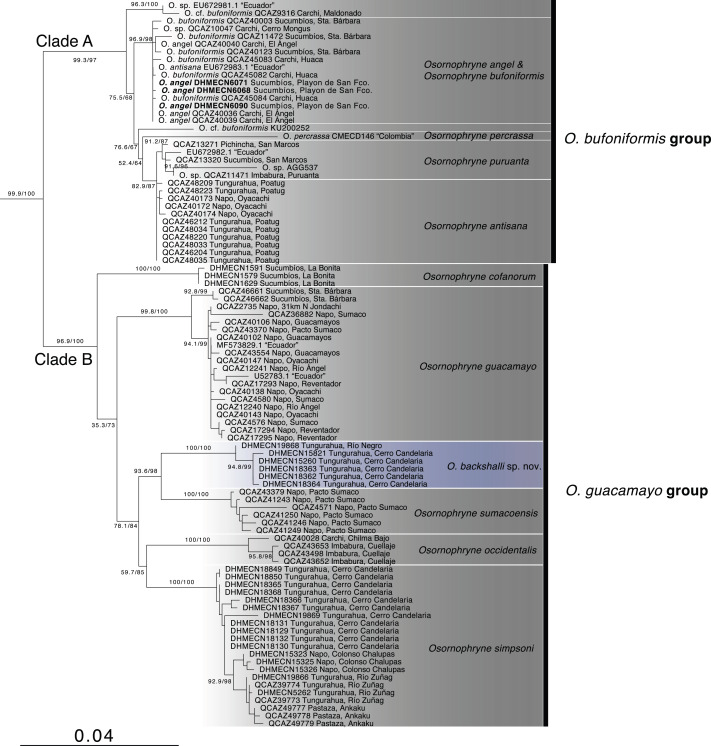
Phylogenetic relationships of *Osornohryne backshalli* sp.nov. with its congeners.

**Phylogenetic relationships** (Fig. 1, Table 1**)**. The partial mitogenome concatenated matrix had 105 terminals and 2146 characters (Appendix S1). IQTree evaluated the best substitution model for the partitions as follows: TIM2+F+G4 for 12S rRNA, TIM2+F+G4 for tRNA-Val, and TIM2+F+I+G4 for 16S rRNA.

Our phylogeny is consistent with previous hypotheses for *Osornophryne* (Páez-Moscoso & Guayasamin, 2012) in delimiting two major clades within the genus: (1) Clade A (*O. bufoniformis* species group) composed of *O. percrassa* + *O. bufoniformis* + *O. angel* + *O. puruanta* + *O. antisana*, and (2) Clade B (*O. guacamayo* species group) composed of *O. cofanorum* + *O. guacamayo* + *O. sumacoensis* + *O. occidentalis* + *O. simpsoni* (Fig. 1).

Our analysis indicates that the new species, *Osornophrye backshalli* sp. nov. belongs to clade B with high support (99.4/100 branch supports). Our inference suggests that the new species is sister to *Osornophryne sumacoensis* (92.6/100 branch supports) and closely related to the subclade *O. simpsoni* + *O. occidentalis* (73.2/87 branch supports).

**Table 1:** Genetic distances between species of the *Osornophryne guacamayo* species group. The number of base differences per site from averaging over all sequence pairs between groups are shown. This analysis involved 60 nucleotide sequences. All ambiguous positions were removed for each sequence pair (pairwise deletion option).

	*O. cofanorum*	*O. guacamayo*	*O. backshalli* sp. nov*.*	*O. sumacoensis*	*O. occidentalis*
*O. cofanorum*					
*O. guacamayo*	4.82342%				
*O. backshalli* sp. nov.	4.57269%	3.47798%			
*O. sumacoensis*	4.58222%	4.30845%	3.30210%		
*O. occidentalis*	6.06343%	4.19563%	4.14410%	5.15024%	
*O. simpsoni*	4.87768%	3.43945%	3.19880%	4.01793%	4.03872%

### Systematics

**Generic placement.** We assign the new species to genus *Osornophryne* based on phylogenetic relationships and external morphology, including extensive webbing between digits and toes that are almost indistinguishable; absence of paratoid glands and auditory structures. In addition, *Osornophryne* species show fused atlas and axis, reduced number of phalanges in hands and feet, sexual dimorphism, and inguinal amplexus (Ruiz-Carranza & Hernández-Camacho, 1976; Hoogmoed, 1987; Gluesenkamp, 1995; Gluesenkamp & Guayasamin, 2008).

### Species accounts

**Table:** 

**New species**
***Osornophryne backshalli*** ** sp. nov.**
***Osornophryne*** sp. nov. INABIO-Instituto Nacional de Biodiversidad (2023)
LSIDurn:lsid:zoobank.org:act:EE7F5D3E-66DE-47D7-B0C1-61FA7D4A7013
***Proposed standard English name. Steve Backshall’s Andean Toad***
***Proposed standard Spanish name. Osornosapo de Steve Backshall***

**Figure 2:**
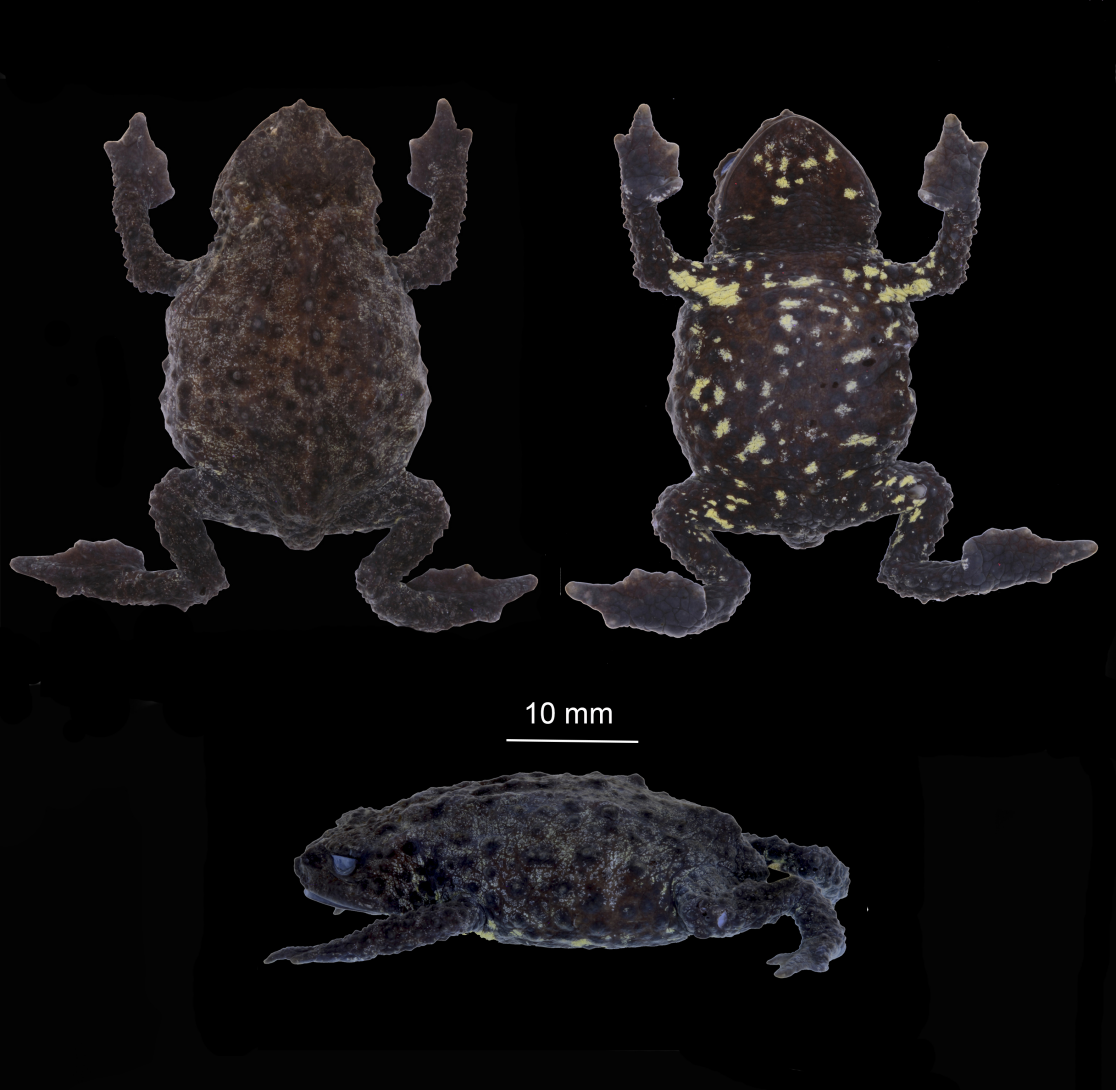
Dorsal ventral and lateral views of preserved *Osornophryne backshalli* sp. nov. holotype (DHMECN 15260), adult female. Photo credit Mario H. Yánez-Muñoz.

**Figure 3:**
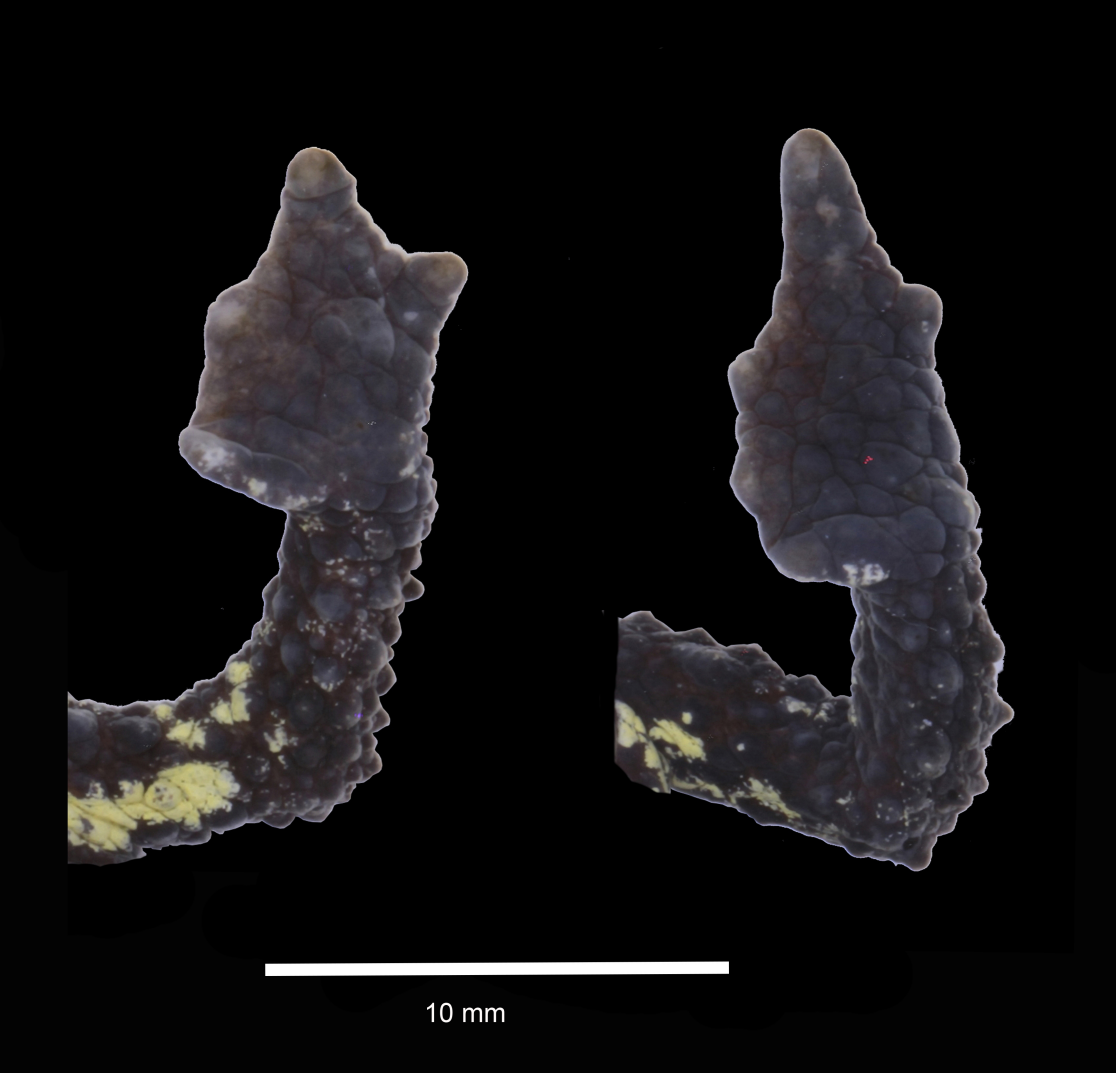
Dorsal and ventral views of hand and foot of preserved *Osornophryne backshalli* sp. nov. holotype (DHMECN 15260), adult female. Photo credit: Mario H. Yánez-Muñoz.

**Figure 4:**
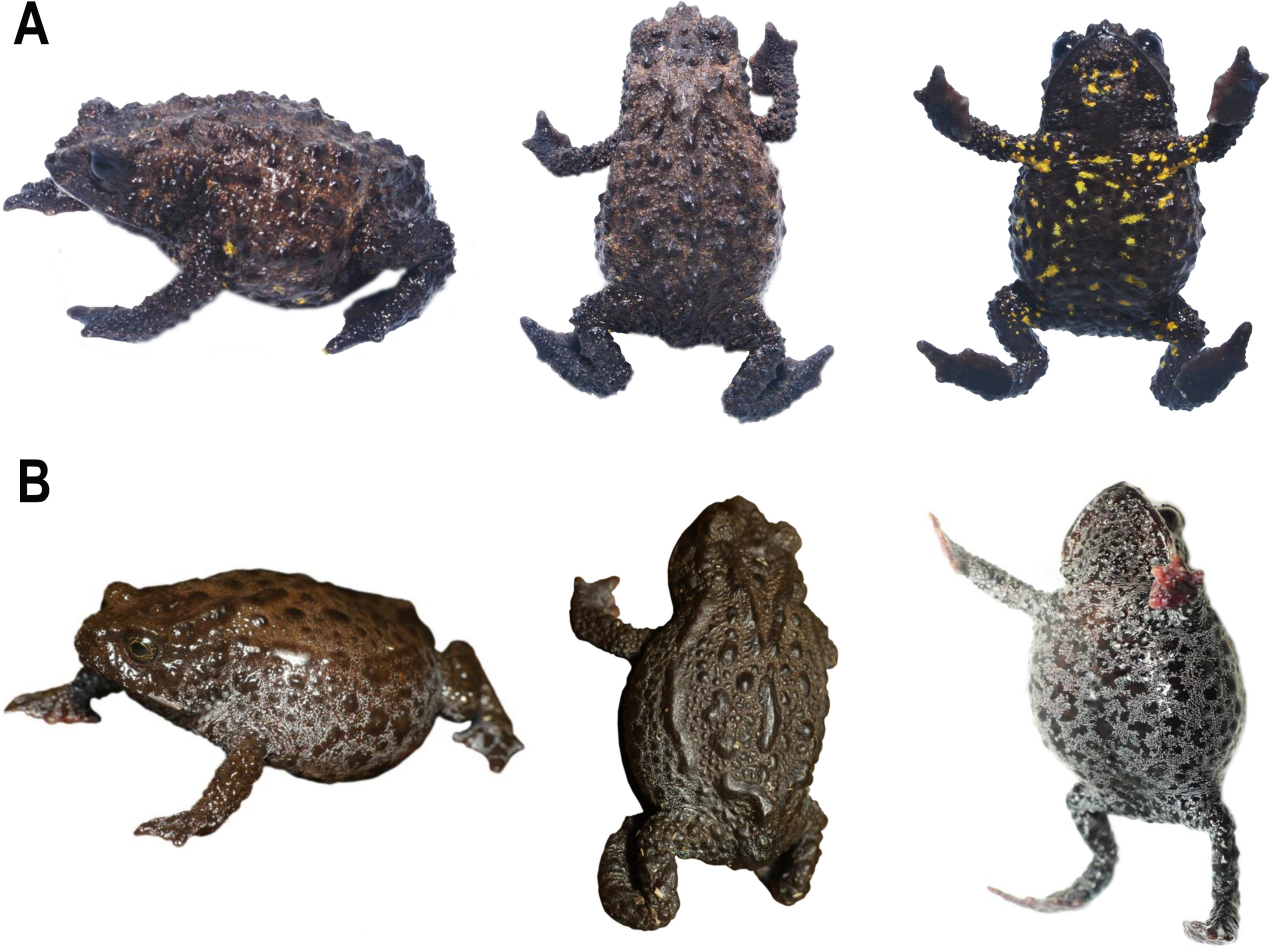
Life comparison of dorsolateral, dorsal and ventral views of female of *Osornophryne backshalli* sp. nov., with its closest relative. (A) *O. backshalli* sp. nov. DHMECN 15260, female holotype from Cerro Candelaria; (B) *O. sumacoensis* MZUTI 6912, female topotype from Sumaco volcano. Photo credit: Mario H. Yánez-Muñoz (A) and Juan P. Reyes-Puig (B).

**Figure 5:**
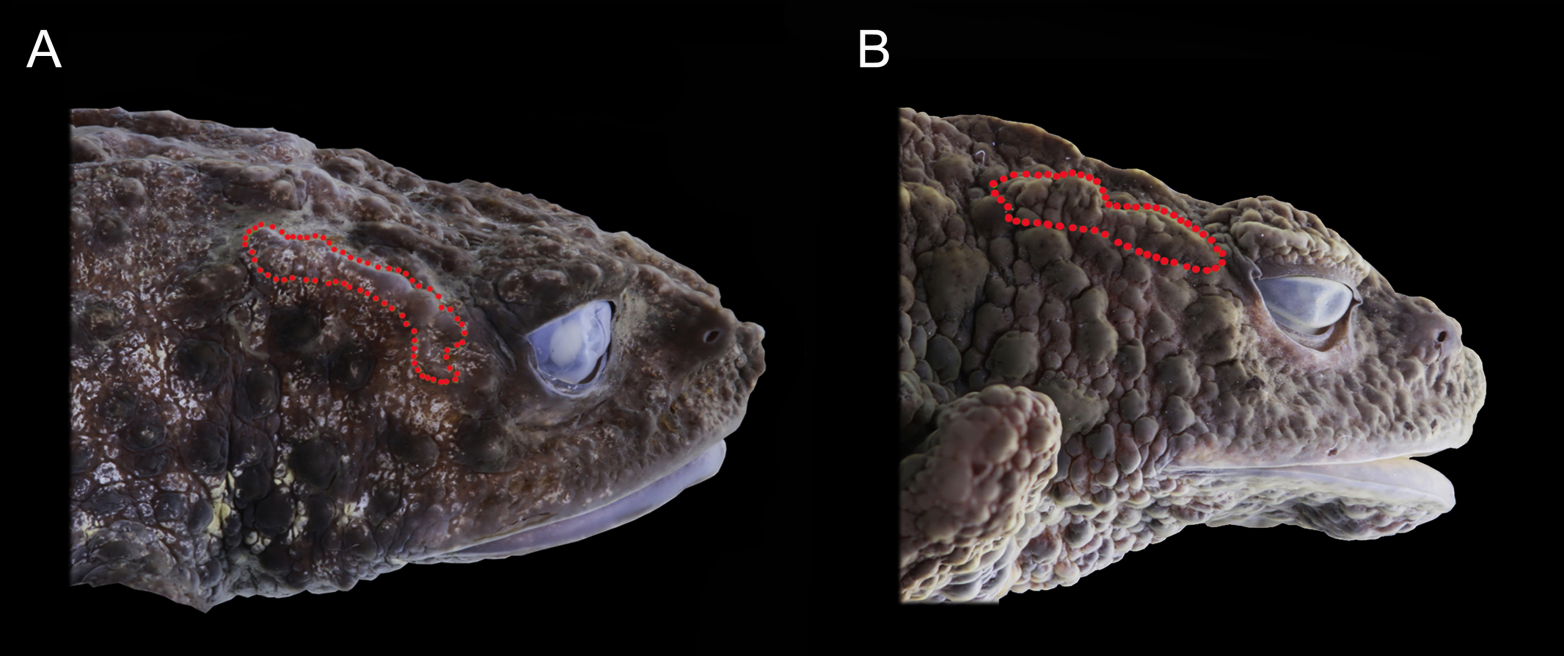
Preserved lateral profile view of *Osornophryne backshalli* sp. nov. in comparison with its closest relative species. Differences in the shape, orientation, and skin texture of the crista parotica are highlighted with red markings. (A) *Osornophryne backshalli* sp. nov., DHMECN15260, female holotype; (B) *O. sumacoensis* QCAZ 4570, female holotype. Photo credit: Mario H. Yánez-Muñoz (A) and Juan P. Reyes-Puig (B).

**Holotype** **(**Figs. 2–5**)**. DHMECN 15260, adult female, from Cerro Candelaria Protected Area, Rio Verde, Tungurahua province, Ecuador, (WGS84 17 M, −1.444600°S, −78.302250°W; 2,725 m), collected on 10 November 2019 by Eduardo Peña & Kelsey Huisman.

**Figure 6:**
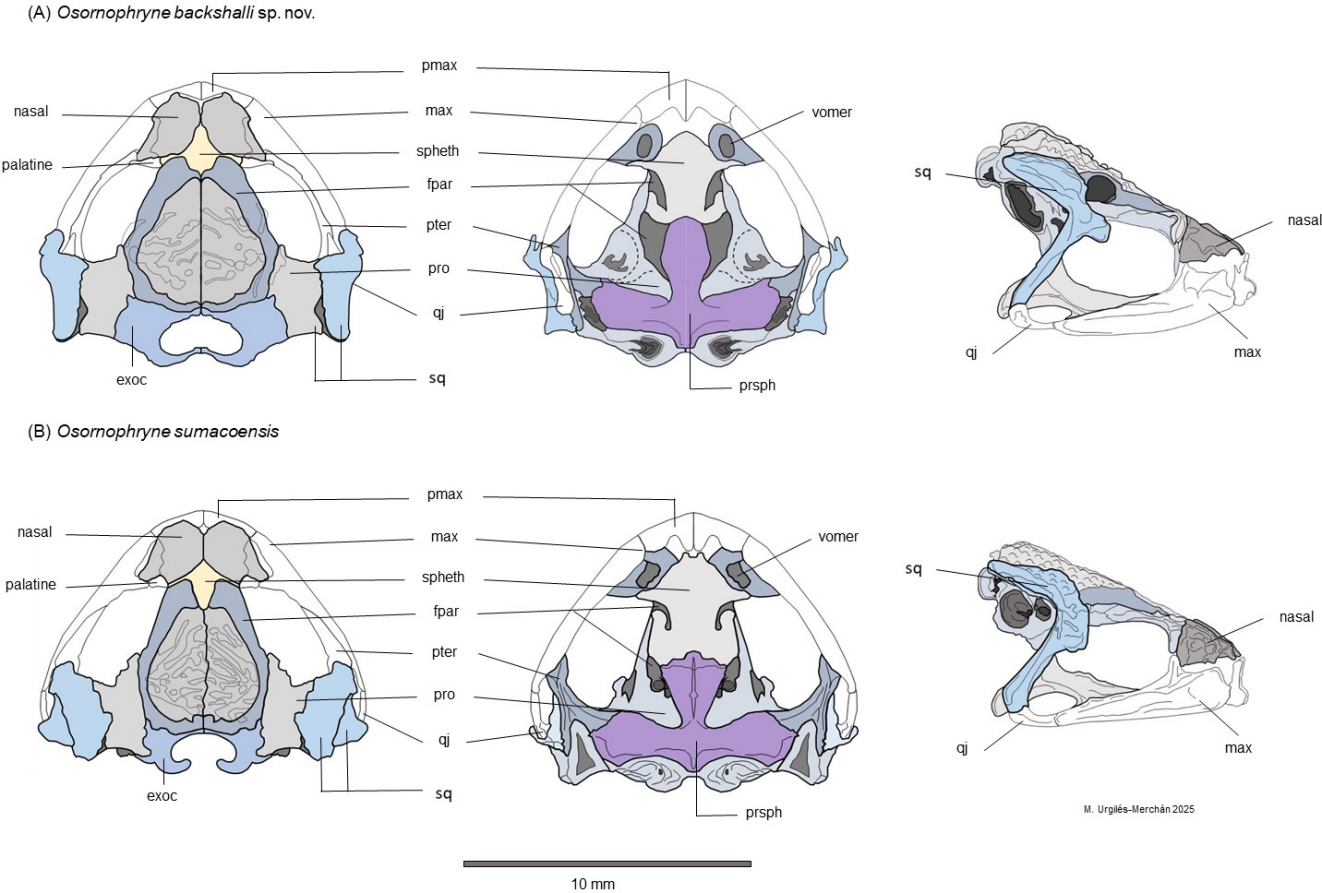
Schematic dorsal, lateral and ventral views of the skull morphology of *Osornophryne backshalli* sp. nov. and its closest relative species *O. sumacoensis*. (A) *Osornophryne backshalli* sp. nov., DHMECN 18363, female paratype; (B) *O. sumacoensis,* MZUTI 5147, female. Diagrams by Miguel Andres Urguilez Merchán. Legend. Abbreviations: pmax, premaxilla; max, maxilla; spheth, sphenethmoid; fpar, frontoparietal; pter, pterygoid; pro, prootic; qj, quadratojugal; sq, squamosal; Exoc, exoccipital; prsph, parasphenoid. Diagrams Miguel A. Urgilés-Merchan.

**Figure 7:**
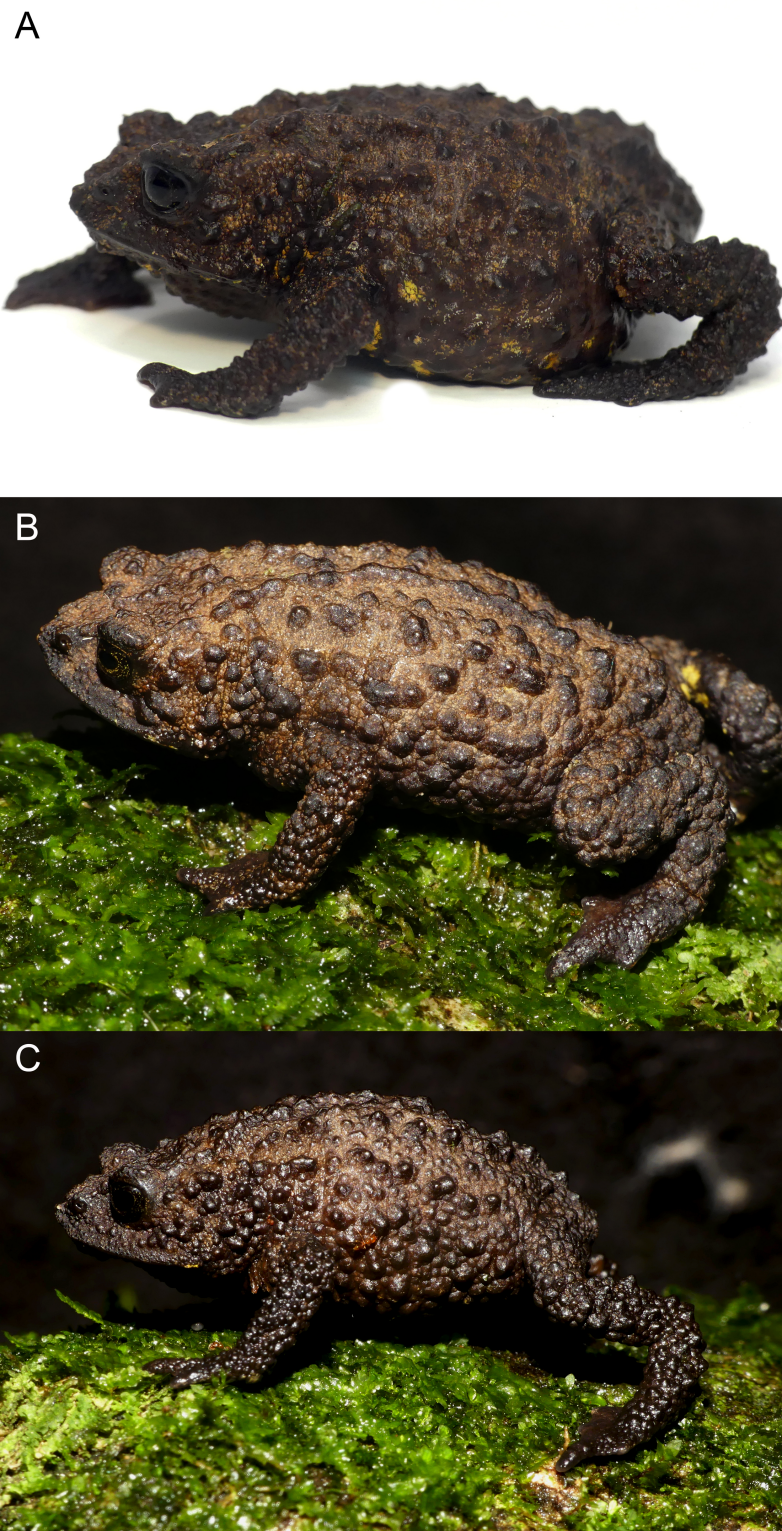
Life coloration and detailed dorsolateral view showing sexual dimorphism of *Osornophryne backshalli* sp. nov. (A) DHMECN 15260, holotype, adult female; (B) DHMECN 18364, paratype, adult female; (C) DHMECN 18364 paratype, adult male. Photo credit: Mario H. Yánez-Muñoz (A) and Juan P. Reyes-Puig (B, C).

**Figure 8:**
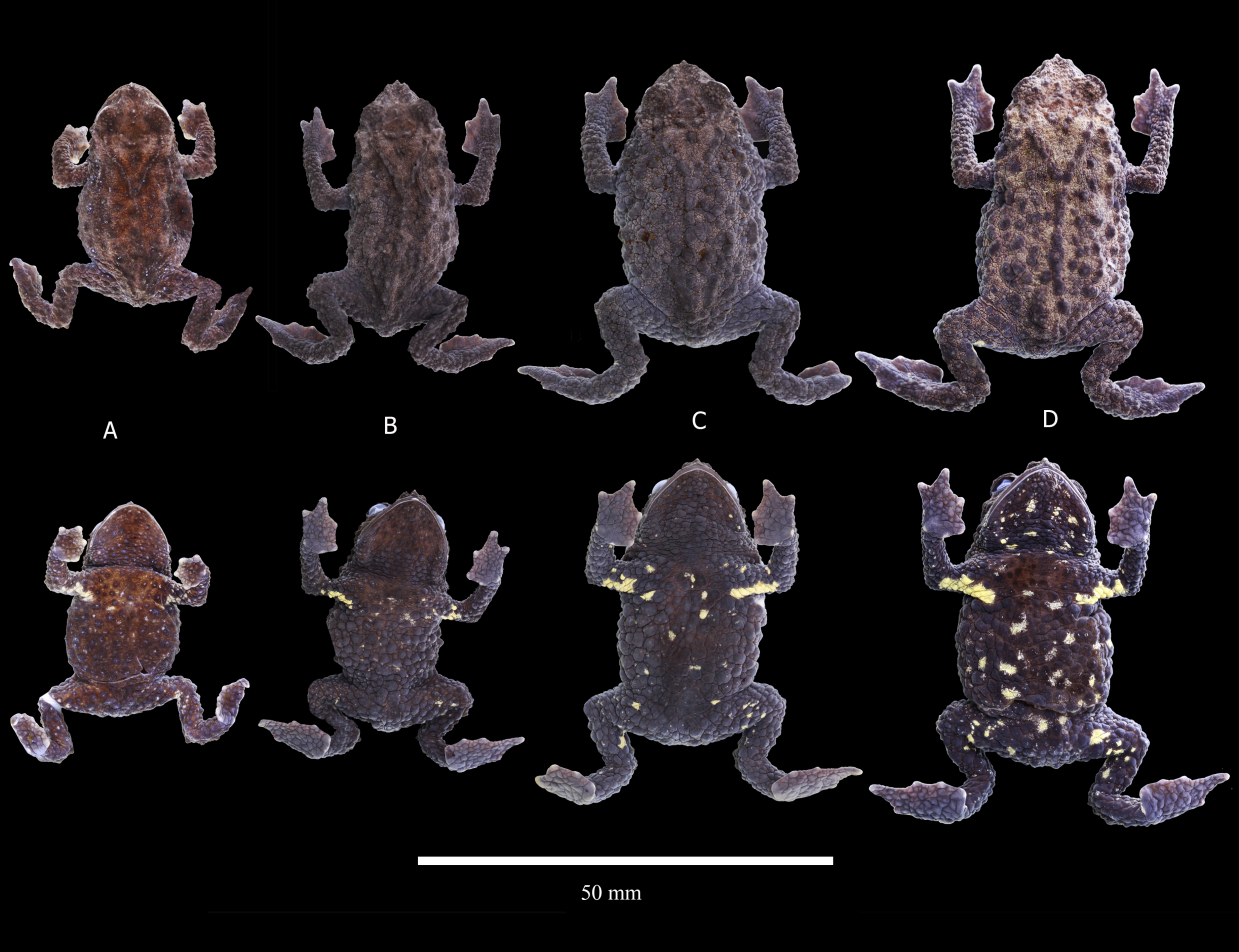
Preserved variation in paratypes of *Osornophryne backshalli* sp. nov. (A) DHMECN 15821, adult male; (B) DHMECN 18364, adult male; (C) DHMECN 18362, adult female; (D) DHMECN 18363, adult female. Photo credit Juan P. Reyes-Puig.

**Figure 9:**
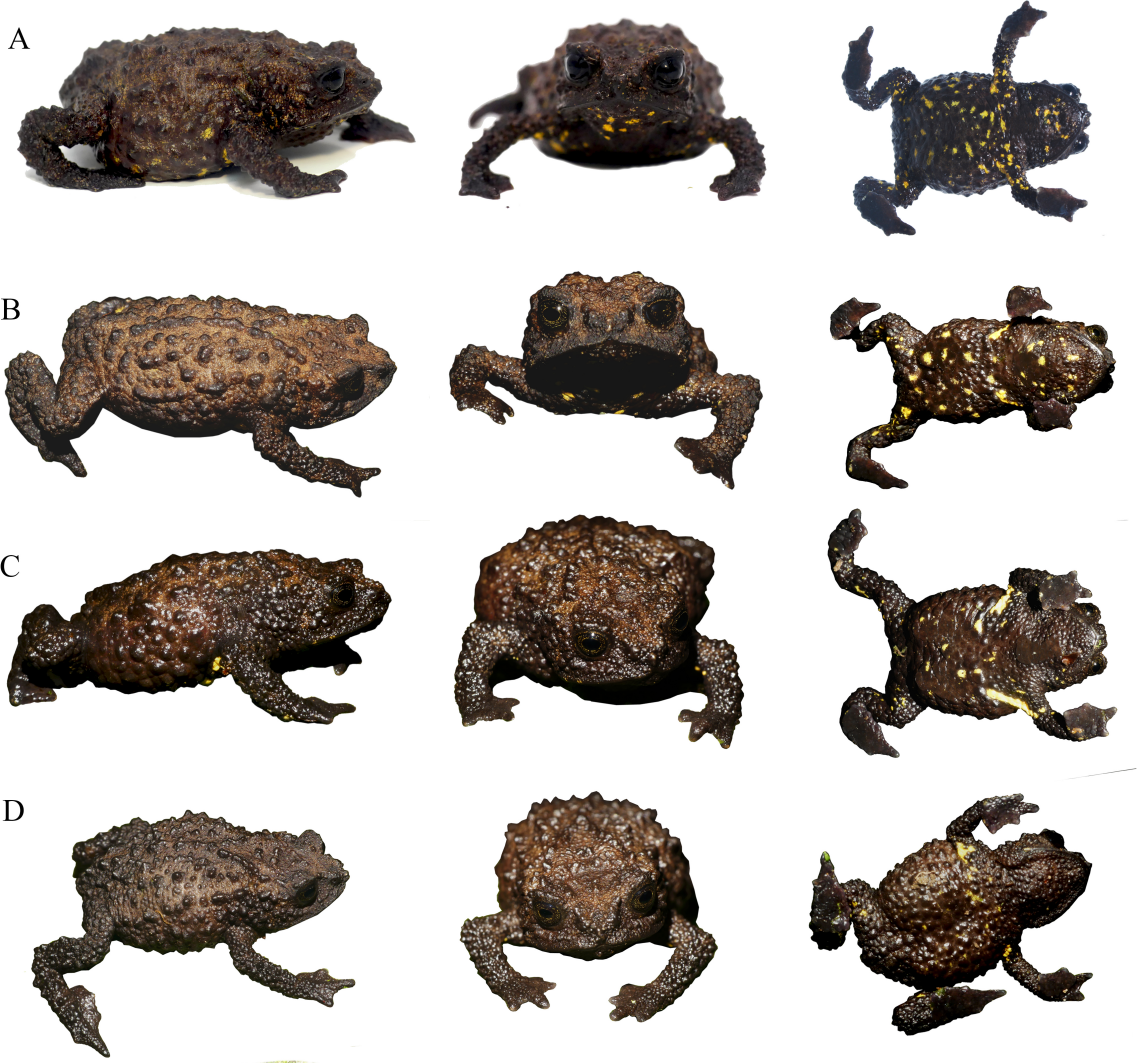
Comparison of the live coloration within the type series of *Osornophryne backshalli* sp. nov. (A) DHMECN 15260, female holotype; (B) DHMECN 18362, adult female paratype; (C) DHMECN 18364, adult male paratype; (D) DHMECN 18363, adult female paratype. photo credit: Mario H. Yánez-Muñoz (A) and Juan P. Reyes-Puig (B, C, D).

**Paratypes** **(**Figs. 6–9**)**. A total of five (5) specimens. Adult females (3): DHMECN 18362, DHMECN 18363, from the same protected area near holotype locality (WGS84 17 M, −1.436783°S, -78.301017°W; 2,583 m) collected on 22 November 2022 by JPRP, Patricio Vinueza, Paulet Benavides & Eduardo Peña; DHMECN 19868, from Finca Palmonte, Rio Negro, Tungurahua Province, Ecuador (WGS84 17 M, −1.438695°S, −78.265438°W; 2,700 m), collected on 15 May 2024 by JPRP, José Ignacio Segovia, Evelyn Toa, Ximena Grefa, Alex Guevara and Fausto Recalde; Adult males (2): DHMECN 15821, same locality as the holotype collected on 14 March 2020 by Fausto Recalde, Juan Pablo Reyes-Puig and Martín Morales; DHMECN 18364, from the same locality and data as DHMECN 18362.

**Diagnosis.** *Osornophryne backshalli* sp. nov. is a member of clade B (*O. guacamayo* species group), and differs from all other species of the clade by the following combination of characters: (1) small to medium size toads (SVL = 22.08–29.68 mm in males, 30.09–35.4 mm on females; Table 2); (2) head slightly wider than long; (3) skin on dorsal surfaces and limbs finely granular with conical and subconical warts, males more tuberculated than females,ventral surfaces with subconic tubercles; (4) snout subacuminate in dorsal view, protruding in lateral profile, with pointed irregular papillae on the tip; (5) crista parotica slightly arched with finely glandular skin, posterior and oblique in relation to the orbit; zygomatic ramus of the squamosal elongated with a blunt anterior border, cultriform process with blunt anterior border; (6) glandular occipital folds present, discontinuous, dorsolateral folds absent, instead a row of discontinuous subconical tubercles on its place; (7) pelvic folds absent (8) limbs short, heels do not touch when adpressed; (9) fingers and toes with extensive and thick webbing, fingers visible, tips of Toes I, II, III almost indistinguishable, Toe V short, not elongated, slightly longer than Toe III, nuptial pads in males on the anterior part of Finger I; (10) dorsal surfaces Brownish Olive with Spectrum Yellow and Light Neutral Gray flecks, ventral surfaces Brownish Olive with Spectrum Yellow blotches; (11) short cloacal tube slightly projected medial to the thighs.

**Comparisons with other species (**Figs. 4–6**).** *Osornophryne backshalli* sp. nov. is distinguished from all other members of the genus by its dorsal and ventral skin texture with subconical and conical tubercles, and also by the Spectrum Yellow blotches on the flanks and belly, and presence of occipital ridge without dorsolateral folds. Other species in eastern Andes, which present at least discontinuous or dorsolateral folds, are *O. simpsoni*, *O.cofanorum O. bufoniformis*, *O. talipes*, *O. angel*, *O. puruanta*, *O. sumacoensis*, and *O. antisana. Osornophryne percrasa* lacks an occipital fold and triangular papilla on the tip of the snout, while the new species has an occipital fold and a dorsolateral row of enlarged tubercles. Also, *Osornophryne backshalli* sp. nov. differs from *O. cofanorum*, *O. guacamayo* and *O. simpsoni* by having reduced Toe V not elongated, while all the latter species bear Toe V barely differentiated from the foot plate (Fig. 4, Table 3).

A systematic comparison of the key diagnostic characters of the new species and its closest congeners is provided in Table 3, with detailed lateral views shown in Figs. 4 and 5. These comparisons highlight the most distinctive external morphological features differentiating *Osornophryne backshalli* sp. nov. from *O. sumacoensis*, its closest known relative.

In lateral profile, *O. backshalli* sp. nov. differs by having a protruding snout bearing large triangular papillae and a smooth, concave loreal region. In contrast, *O. sumacoensis* exhibits a rounded snout with rounded papillae and a flat, tuberculate loreal region. The dorsal surface of the head in the new species is covered by elevated subconical tubercles, whereas in *O. sumacoensis* it is covered by rounded warts. The *crista parotica* in *O. backshalli* sp. nov. is slightly arched, positioned posterior and oblique relative to the orbit, and covered by finely glandular skin. In contrast, in *O. sumacoensis* it is more horizontally aligned and posterior to the orbit, and covered with dense, rounded warts (Fig. 5).

Additionally, *O. backshalli* sp. nov. bears a dorsolateral row of enlarged conical tubercles, whereas *O. sumacoensis* has a less prominent pustular ridge. The forearms of the new species present large subconical tubercles, in contrast to the rounded tubercles in *O. sumacoensis*. On the dorsum, *O. backshalli* sp. nov. exhibits elevated subconical tubercles, whereas *O. sumacoensis* has a granular texture interspersed with rounded warts. The palms and soles in the new species are smooth, while in *O. sumacoensis* they are covered by flat, rounded supernumerary tubercles. Finally, the cloacal tube of *O. backshalli* sp. nov. is more elongated and ventrally directed, in contrast to the shorter, less protruding structure in *O. sumacoensis* (Fig. 4).

The skull of *O. backshalli* sp. nov. presents differences from *O. simpsoni* and *O. sumacoensis* in the shape of the sphenethmoides in dorsal view (see Fig. 6, Table 4 and (Páez-Moscoso, Guayasamin & Yánez-Muñoz, 2011). In *O. backshalli* sp. nov. the anterior border is elongated and projected compared to the posterior border, which is short, irregular, and not projected posteriorly. Contrary to the acuminate short anterior border of the sphenethmoides in *O. sumacoensis*, the new species has a posterior elongated border extending into the interparietal suture. Additionally, the shape of the zygomatic ramus of the squamosal presents distinctive differences (Fig. 6), with a more projected and blunt anterior border, extending to the level of the third portion of the maxillae in *O. backshalli* sp. nov., contrary to the short, broad, and not projected anterior border of the zygomatic ramus in *O. sumacoensis*. Otic ramus is slightly arched at the suture with the crista parotica in *O. backshalli* sp. nov., while the otic ramus is enlarged posteriorly and slightly oblique at the suture of the crista parotica in *O. sumacoensis*. Cultriform process is longer in *O. backshalli* sp. nov., than in *O. sumacoensis* (Fig. 6, Table 4).

**Table 2:** Morphometric character measurements in millimeters (mm.), for the type series of *Osornophryne backshalli* sp. nov. For abbreviations, see the ‘Methods’. Parenthesis in Ranke columns (x = mean/sd = standard desviation).

***Osornophryne backshalli*** ** sp.nov**								
**Character**	**Males**		**Females**				**Ranke males (2)**	**Ranke females (4)**
	**DHMECN**		**DHMECN**					
	**15821**	**18364**	**15260**	**18362**	**18363**	**19868**		
**SVL**	22.08	29.68	35.4	32.69	30.09	32.47	22.08–29.68 (25.88 ± 5.37)	30.09–35.4 (32.75 ± 3.75)
**TIB**	7.01	9.11	9.74	9.86	8.67	9.83	7.01–9.11 (8.06 ± 1.48)	8.67–9.86 (9.27 ± 0.84)
**FL**	5.96	9.65	10.85	10.75	9.9	9.7	5.96–9.65 (7.81 ± 2.61)	9.7–10.85 (10.28 ± 0.81)
**HaL**	4.91	6.9	7.62	7.6	6.72	7.28	4.91–6.9 (5.91 ± 1.41)	6.72–7.62 (7.17 ± 0.64)
**HL**	7.15	9.69	10.9	10.33	9.18	10.03	7.15–9.69 (8.42 ± 1.8)	9.18–10.9 (10.04 ± 1.22)
**HW**	8.23	10.98	12.71	12.05	11.13	11.69	8.23–10.98 (9.61 ± 1.94)	11.13–12.71 (11.92 ± 1.12)
**IOD**	2.36	3.31	3.44	3.58	3.42	2.58	2.36–3.31 (2.84 ± 0.67)	2.58–3.58 (3.08 ± 0.71)
**EW**	1.79	2.4	2.73	2.8	2.68	2.52	1.79–2.4 (2.1 ± 0.43)	2.52–2.8 (2.66 ± 0.2)
**IND**	2.72	3.18	3.64	3.42	2.98	3.36	2.72–3.18 (2.95 ± 0.33)	2.98–3.64 (3.31 ± 0.47)
**EN**	1.54	2.01	2.17	2.15	1.81	1.7	1.54–2.01 (1.78 ± 0.33)	1.7–2.17 (1.94 ± 0.33)
**SE**	3.07	4.19	4.52	4.33	3.9	4.02	3.07–4.19 (3.63 ± 0.79)	3.9–4.52 (4.21 ± 0.44)
**ED**	2.35	2.9	3.35	3.2	2.54	3.16	2.35–2.9 (2.63 ± 0.39)	2.54–3.35 (2.95 ± 0.57)
**FIIIL**	4.2	5.99	6.98	6.83	5.78	6.33	4.2–5.99 (5.1 ± 1.27)	5.78–6.98 (6.38 ± 0.85)
**FIVL**	3.1	4.53	4.95	4.81	4.33	4.44	3.1–4.53 (3.82 ± 1.01)	4.33–4.95 (4.64 ± 0.44)
**TIVL**	5.6	9.2	10.05	9.79	8.97	9.34	5.6–9.2 (7.4 ± 2.55)	8.97–10.05 (9.51 ± 0.76)
**TVL**	3.87	6.36	6.53	6.45	5.9	6.1	3.87–6.36 (5.12 ± 1.76)	(5.9–6.53) 6.22 ± 0.45

**Table 3:** Comparative morphological traits distinguishing *Osornophryne backshalli* sp. nov. from closely related species in the *O. guacamayo* species group, all sharing a reduced Toe V. Characters based on Páez-Moscoso & Guayasamin (2012) and this work.

Species	Dorsolateral fold/ridge	Occipital folds	Pelvic folds	Flanks texture	Dorsum	Head in dorsal view	Head in profile	Toea III–V condition	Life dorsal coloration	Life ventral coloration
*Osornophryne backshalli* sp. nov.	Absent, row of subconical tubercles discontinuous	Glandular dermic folds forming a “V” extending into the sacrum	Absent	Subconic warts with small tubercles in males than in female	Subconic warts with small tubercles in males than in female	Rounded with a triangular papilla on the tip of the snout/	Truncate with triangular papilla	Toes reduced with exception of Toe IV; Toe V reduced slightly longer than III/	Brownish Olive with Spectrum Yellow and Light Neutral Gray grey fleck	Brownish Olive with Spectrum Yellow bright blotches.
*Osornophryne sumacoensis*	Present pustular ridge	Present continuous or discontinuous	Present	Dominated by relatively small pustules	Dominated by relatively small pustules, with scattered large pustules	Males: Subacuminate, with papilla or very short proboscis. Females: subacuminate, with papilla at tip	Males: Subacuminate to acuminate, with papilla or very short proboscis, Females: Round to subacuminate, with papilla at tip	Reduced, V same length than III	Brown to orange brown, Females dark brown	Males pale brown to orange brown; Females: Pale grayish blue, with dark gray to black blotches
*Osornophryne occidentalis*	Discontinuous composed of small warts	Discontinuous composed of small warts	Low or absent	Dominated by numerous warts with scattered medium size pustules	Covered of pustules of different sizes providing a wrinkled texture	Acuminated with papilla at tip	Acuminated to subacuminated with papilla at tip	Not reduced, Toe V elongated longer than toe III, and reach medial portion of toe IV	Olive green to Dark brown to black, with or without a cream to light brown dorsolateral stripes.	Males: Light brown to dark brown with few yellow pustules
*Osornophryne simpsoni*	Present, discontinuous, composed of small	Present, discontinuous, composed of small	Absent	With numerous small warts and scattered conical tubercles	With numerous small warts and scattered conical tubercles	Truncate to round, with papilla	Truncate to round, with papilla at tip	Elongated, extending towards medial portion of Toe IV	Light Brown to dark brown	Grayish brown to dark brown, with cream to yellow pustules

**Table 4:** Measurements of the skull and osteological diagnostic characters of *Osonophryne backshalli* sp. nov., in comparison with its sister species *O. sumacoensis*.

Species	Skull width (mm)	Skull length (mm)	Braincase width (mm)	Parasphenoid alae width (mm)	Cultriform proccess length (mm)	Shape of squamosal	Shape of sphenethmoid	Shape of parasphenoid
*Osornophryne backshalli* sp. nov.	10.35	9.5	3.36	3.24	3.69	Zygomatic ramus elongated with blunt anterior border projected, otic ramus arched	Anterior process subacuminated and projected anteriorly at the middle level of nasals, posterior process of sphenethmoid is short and slightly subacuminated	Cultriform process with blunt anterior border
*Osornophryne sumacoensis*	10.34	9.62	2.95	3.24	3.39	Zygomatic ramus short and broad anterior border not projected, otic ramus oblique	Anterior process short and acuminated, posterior process elongated	Cultriform process short and broad with irregular anterior border

**Description of the holotype** **(**Figs. 2–3**)**. DHMECN 15260, adult female, moderate in size, 35.4 mm SVL. Head length 85.7% head width; width of head greater at level of posterior margin of mouth; snout protuberant, with rostral triangular papillae in dorsal and lateral views; nostrils rounded, protuberant, laterally oriented; each nostril oblique and oval, frontally projected; internarial area rough and slightly concave; interorbital region tuberculatewith scattered conic tubercles, skin co-ossified with underlying bones; upper eyelids strongly tuberculate bearing several subconic warts; interorbital region wider than the upper eyelid (79.4%), interorbital subconic tubercle present; outer edge of the eyelid delineated by a continuous row of warts; canthus rostralis straight and tuberculated; loreal region concave and rugose; eyes with elliptically horizontal pupil; infraorbital and postorbital regions rugose with subconic warts posteriorly; occipital surface rough with subconic warts and two low glandular ridges forming a “Y”, converging posteriorly in middle dorsal line (Fig. 2); crista parotica forming an elevated glandular ridge located posterior and oblique to the orbit. Skin of dorsum and flanks finely granular, with numerous subconic and conic warts; without dorsolateral ridge, in its place a row of elongated subconic tubercles, pelvic ridge absent; limbs tuberculate with subconic elongated warts; ventral skin with subconic tubercles in the belly and chest, becoming more elevated towards the flanks and limbs. Forelimbs short, slender, strongly tuberculated with rows of subconical tubercles in the fore arms; hand moderate in length, representing 21.5% of SVL; extensive webbing between fingers (Fig. 3); lengths of fingers in order of increasing length: I = II < IV < III; palms with merged flattened tubercles, subarticular tubercles not evident; large irregular palmar tubercle slightly differentiated, thenar tubercle oval.

Hind limbs short, slender, and covered dorsally with large subconical tubercles, tibia and foot, respectively, 27.5% and 30.6% of SVL; extensive thick skin between toes, webbing between Toes I–III,V more extensive that webbing between Toes IV (Fig. 3); lengths of Toes: I < II < III < V< IV; Toe not differentiated and longer than Toe III, soles with flattened tubercles; subarticular tubercles not evident; inner metatarsal tubercle irregular. Skin on the groin less tuberculate in the femoral region. Choanae small, rounded, widely separated; cloacal opening medial to thighs, slightly projected, with short cloacal tube present.

**Color of holotype in life** **(**Figs. 4, 7, 9**)**. Dorsal surfaces of head, snout and body Brownish Olive (276) with small marks Spectrum Yellow (79), warts on dorsum and flanks Hair Brown (277), limbs darker. Iris Dusky Brown (285) with Dark Spectrum Yellow (78) punctuations. Belly and ventral surfaces of the body including thighs, chest, shoulders, throat and chin Olive Brown (278) with Spectrum Yellow (79) irregular blotches and marks. Palms and soles dark Raw Umber (280).

**Color of holotype in ethanol 70%.** **(**Fig. 2**)**. Dorsal surfaces Brownish Olive (276) with small Tawny Olive (17) light reticulations in the background, warts and tubercles Olive Brown (278). Belly Vandyke Brown (282) with scattered Pale Buff (1) blotches, surfaces of palms and soles Brownish Olive (276) with some scattered Tawny Olive (17) marks.

**Osteologic diagnostic characters of the Skull (**Fig. 6**)**. The skull of the adult female paratype (DHMECN 18363) is illustrated in dorsal, lateral, and ventral views (Fig. 6). Based on previous osteological descriptions of *Osornophryne* (Ruiz Carranza & Hernández, 1976; Páez-Moscoso, Guayasamin & Yánez-Muñoz, 2011), we summarized the osteologic diagnostic characters of the new species in Table 4. We describe only those skull bones that exhibit diagnostic differences.

The cranium is widest posterior to the orbit at the level of the quadratojugal, with a broad braincase. The braincase width, measured at the mid-orbit level, represents approximately 32.4% of the greatest skull width and 35.4% of the medial skull length.

Among the neurocranial bones, key diagnostic characters are observed in the sphenethmoid, squamosal and parasphemoid. Dorsally, sphenethmoid presents an anterior subacuminate process, reaching the middle level of the nasals, and a short posterior and slightly subacuminate process is evident as well. In ventral view, the anterior process of the sphenethmoid is slightly acuminate and reaches the posterior level of the choanae, and is separated from premaxilla by paired nasals.

In lateral view, the shape and projection of the squamosal components are also diagnostic. The otic ramus is slightly arched and broad, forming a prominent crista parotica. Additionally, the zygomatic ramus of the squamosal shows an anterior elongation with a blunt border that reaches the level of the third part of the maxillae. The frontoparietal fontanelle is not evident; instead, the frontoparietal suture is clearly visible. The exoccipitals are nearly in contact but slightly separated medially, epiotic eminences are not prominent and the dorsal surface of the prootics is smooth.

In the ventral view, the skull exhibits the parasphenoid with its characteristic inverted T shape. The cultriform process extends anteriorly just behind the mid-level of the orbit, contacting the posterior process of the sphenethmoid. The cultrifrom process is blunt on its anterior margin and reaches its maximum width at the level of the posterior border of the optic fenestra. The parasphenoid alae are robust, with irregular lateral margins that articulate with the pterygoids and posteriorly with the prootic and paired exoccipitals. Each alae measures 87.8% of the length of the cultriform process. The posterior margin of the parasphenoid bears a short, truncated posteromedial process that terminates just anterior to the margin of the foramen magnum (Fig. 9).

**Variation** (Figs. 7–9). *Osornophryne backshalli* sp.nov. is a sexually dimorphic species with nuptial pads on males, and smaller SVL in males than in females; measurements of the type series are presented on Table 3. There is little color variation, with slight variation in the brown and yellow tones on dorsal and ventral surfaces (Figs. 7, 9). The male paratype DHMECN 15821 presents Brownish Olive (276) dorsal surfaces of body and head with Light Yellow Ochre (13) tones and Verona Brown warts, and the tip of the snout presents a Light Yellow Ochre (13) wart. Iris is Dusky Brown (285) with Dark Spectrum Yellow (78) dark reticulations. Belly and ventral surfaces of limbs, including palms and soles, Dark Grayish Brown (284) with Spectral Yellow (79) small marks and blotches. In general, the other specimens present similar color patterns, with the exception of the shape and size of the bright yellow ventral pattern (Figs. 8–9).

**Figure 10:**
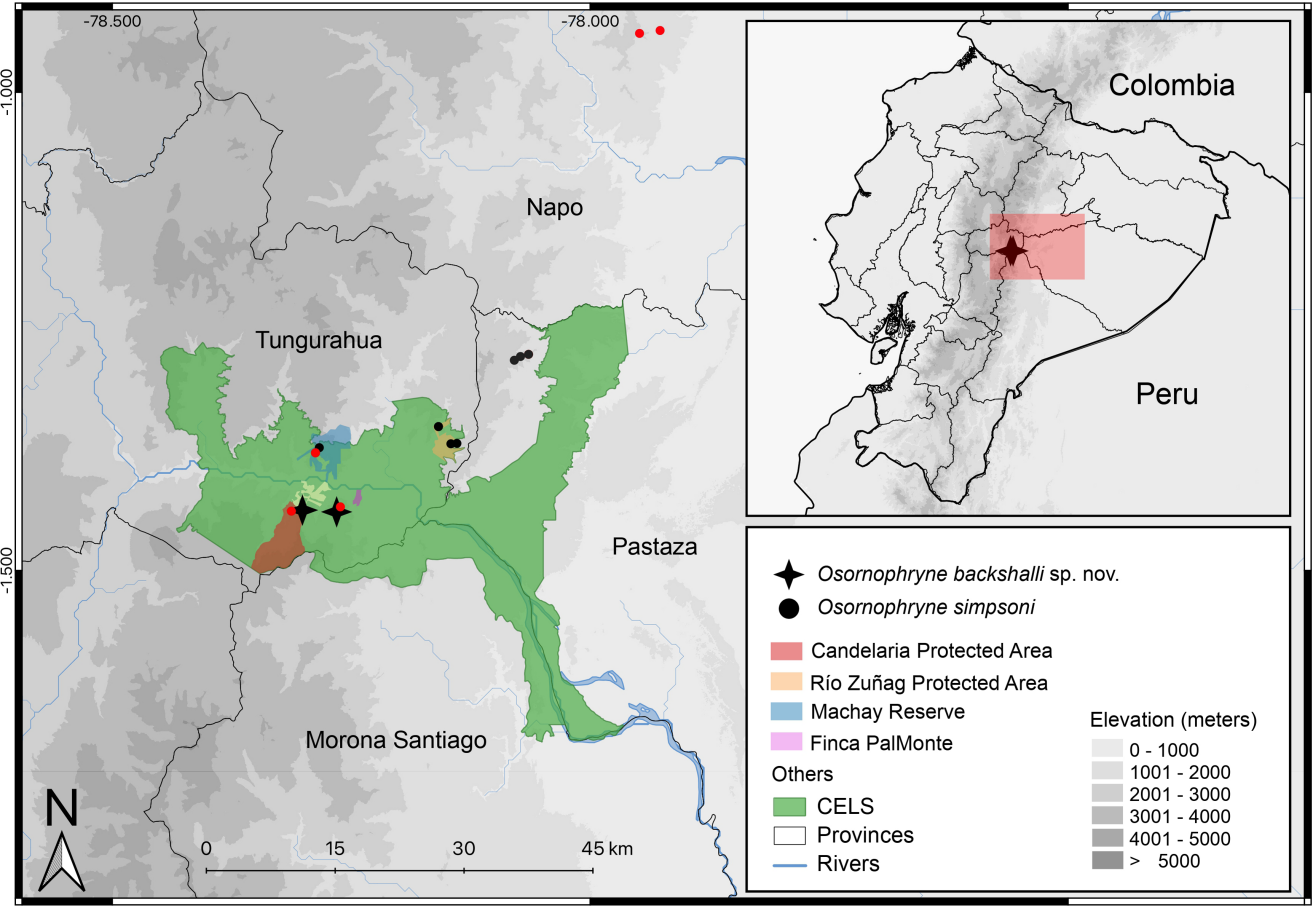
Distribution map of *Osornophryne backshalli* sp. nov., and new records of *Osornophryne simpsoni* in red. Green area correspond to the “Corredor Ecológico Llanganates-Sangay (CELS)”. Map by Julio C. Carríon.

**Distribution and natural history** (Fig. 10). *Osornophryne backshalli* sp. nov. is currently known only from two localities on the northern and eastern slopes of Cerro Candelaria, Rio Verde and Rio Negro Parish, Baños Township, Tungurahua Province, Ecuador (Fig. 10), at elevations ranging from 2,568 to 2,725 m. These sites are located within montane cloud forest, classified as “Bosque Siempreverde Montano del Norte y Centro de la Cordillera Oriental de los Andes” (Ministerio del Ambiente del Ecuador, 2013). The forest canopy reaches approximately 20 m in height and is dominated by palm trees (*Ceroxylum* spp.), along with *Clusia* and *Weinmannia* trees, all of which are densely covered by bryophytes and epiphytes. The understory features numerous fallen logs and a thick layer of leaf litter on the forest floor.

The holotype was found during the day on a moss-covered fallen trunk, approximately 50 cm above the ground. The male paratype (DHMECN 15821) was observed at night on branches of moss-covered shrubs, about 130 cm above the ground. The remaining paratypes were encountered at night within the leaf litter on the forest floor.

Amphibian species found in sympatry with *O. backshalli* sp. nov. include *Osornophryne simpsoni*, *Pristimantis tungurahua*, *P. marcoreyesi*, *P. ardyae*, *P. donnelsoni*, and *Niceforonia* sp. Despite several targeted herpetological surveys conducted over the past sixteen years in Cerro Candelaria, with a particular focus on documenting local amphibian diversity, fewer than six individuals of *O. backshalli* sp. nov. have been recorded, suggesting that the species is relatively rare and has a low population density.

**Etymology.** The specific epithet is a patronym in honor of the explorer and television presenter Steve Backshall, of London, UK. He has raised awareness of nature around the world and has, through his patronage of the World Land Trust, contributed directly to the conservation of the Corredor de Conectividad Llanganates-Sangay, the habitat of this particular species and many others.

**Conservation.** The two localities where *Osornophryne backshalli* sp. nov. occurs are inside protected areas. Cerro Candelaria, a reserve owned by the Ecuadorian NGO Fundación EcoMinga, was declared as Private Protected Area by the Ecuadorian Government in 2023, and Finca Palmonte is a private reserve and Socio Bosque area managed by a local family; both areas are on the northern and eastern slopes of Cerro Candelaria, and are part of the buffer zone of Sangay National Park and the Corredor de Conectividad Llanganates Sangay, recently declared by national authorities. Based on the small number of records of the species and its cryptic coloration, we suggest classification under IUCN Red list criteria as Data Deficient DD Category. Future assessments may confirm its small distribution range and specific habitat requirements; the species may then qualify under a threat category.

## Discussion

Our phylogenetic analysis strongly supports the existence of two well-defined clades within *Osornophryne,* corroborating the findings of Páez-Moscoso & Guayasamin (2012). The first clade, here designated as the *O. bufoniformis* species group, includes five species inhabiting high Andean ecosystems in Ecuador and Colombia. These are characterized by their terrestrial-semi-fossorial habits, compact body forms, and a reduced Toe V (shorter than Toes I–III). Despite low interspecific genetic divergence, members of this clade exhibit distinct morphological and morphometric traits.

The second clade, the *O. guacamayo* species group, comprises six species distributed mainly across the humid montane forests on the eastern (and to a lesser extent western) slopes of the Ecuadorian Andes. This group displays greater genetic divergence and morphological variability, and contains two distinct ecotypes. The first includes small, semifossorial toads with reduced Toe V (*O. sumacoensis, O. backshalli sp. nov., O. occidentalis*), while the second features more slender, terrestrial-shrub-dwelling species with elongated Toe V (*O. guacamayo, O. cofanorum, O. simpsoni*). Although previous studies proposed Toe V as a phylogenetically informative character, our data indicates that its variation is homoplasious, representing the result of convergent adaptation to habitat rather than shared ancestry.

Field observations in Cerro Candelaria and Sumaco revealed sympatric occurrences of both ecotypes. Species with reduced Toe V (*O. backshalli* sp. nov. and O*. sumacoensis*) are infrequent and found within a narrow elevational band (2500–2800 m), primarily on the forest floor. In contrast, species with longer Toe V, such as *O. guacamayo* and *O. simpsoni*, are more commonly observed in low shrubby vegetation (ferns and shrubs up to 150 cm high) within forest.

Our findings extend the known distribution of the genus southward beyond the Río Pastaza for the first time (Fig. 10), a significant range extension from previous records limited to areas north of the valley (Páez-Moscoso, Guayasamin & Yánez-Muñoz, 2011).

We report new populations of *O. simpsoni* at both sides of the Pastaza, extending its known range towards the southern and west (DHMECN 18365–18370 on Cerro Candelaria, and DHMECN 18128–18132 at Finca Palmonte, both in Tungurahua province between 2,405 m and 2,583 m), and to the north (DHMECN 15324–DHMECN 15338 in the Reserva Biológica Colonso Chalupas, Napo province, between 2,196 m and 2,539 m). Morphological variation is evident in the geographic extremes of *O. simpsoni,* especially in the shape of the snout, loreal region, and crista parotica.

Osteological comparison between *O. sumacoensis* and *O. backshalli* sp. nov. reveals paired nasals in median contact and solid frontoparietals without fontanelle in both species. However, notable differences exist in the shape and articulation of the anterior and posterior process of sphenethmoid, shape and size in the squamosal components (especially the zygomatic ramus), and parasphenoid (Fig. 6, Table 4). These traits are taxonomically informative and enhance species delimitation within the genus.

While studies of *Osornophryne* skull osteology remain scarce, our contribution includes the first key character descriptions and diagrams of the *O. sumacoensis* skull (MZUTI 6912). We highlight the taxonomic value of features such as the orientation of the crista parotica (formed by the otic ramus of the squamosal), the anterior margin of the zygomatic ramus of squamosal, and parasphenoid morphology (Fig. 6). These characters should be prioritized in future taxonomic assessments.

Advanced imaging methods such as computed tomography (CT) scanning, used successfully in other amphibian taxa such as *Hyloscirtus*, and *Noblella* (Reyes-Puig et al., 2019; Reyes-Puig et al., 2022), would greatly enhance osteological resolution in *Osornophryne* genus. However, until such tools are routinely available, traditional morphological approaches continue to provide valuable insights into cranial anatomy and species boundaries.

## Conclusions

This study not only formally describes a new species (*O. backshalli* sp. nov.) but also contributes to refining the phylogenetic framework of *Osornophryne*, which now comprises 12 recognized species in the northern Andes. The identification of two geographically and ecologically distinct clades suggests divergent evolutionary histories influenced by Andean topography and habitat specialization. Importantly, our data confirm that the Río Pastaza does not represent a strict biogeographic barrier, challenging previous assumptions and highlighting the need for further exploration in under-sampled southern regions.

Additionally, we demonstrate that skull morphology, particularly traits of the sphenethmoid, squamosal, and parasphenoid, offers key characters for species-level diagnosis. These osteological characters warrant inclusion in future integrative taxonomic frameworks, especially as CT-based anatomical datasets become more accessible. Overall, our findings underscore the evolutionary and conservation significance of *Osornophryne* diversity in the Andes and emphasize the importance of continued fieldwork and morphological study.

## Supplemental Information

10.7717/peerj.19760/supp-1 Supplemental Information 1 GenBank Accession numbers for DNA sequences used for phylogenetic analyses

10.7717/peerj.19760/supp-2 Supplemental Information 2 Examined specimens

## Additional Information and Declarations

### Data Availability

The following information was supplied regarding data availability: The sequences are available at Genbank: PV179439 to PV179479.

### New Species Registration

The following information was supplied regarding the registration of a newly described species: Publication LSID: urn:lsid:zoobank.org:pub:459B3032-0A5E-4BDA-8457-9D4D53885725 Species name LSID: urn:lsid:zoobank.org:act:EE7F5D3E-66DE-47D7-B0C1-61FA7D4A7013
