# Supplementary material for: New Andean plump toad of the genus Osornophryne (Anura: Bufonidae) from Cerro Candelaria, Ecuador
Source: PeerJ. 2025 Jul 23;13:e19760. doi: 10.7717/peerj.19760 (PMC12296580; doi:10.7717/peerj.19760)
Supplement: Supplemental Information 1 [file peerj-13-19760-s001.docx]

| *Species* | Citation | Voucher number | 12S rRNA | 16S rRNA |
| --- | --- | --- | --- | --- |
| *Osornophryne angel* | Yánez-Muñoz et al. (2010) | QCAZ40036 | JF907458.1 | JX411998.1 |
| *Osornophryne angel* | Yánez-Muñoz et al. (2010) | QCAZ40039 | JF907459.1 | JX411999.1 |
| *Osornophryne angel* | Yánez-Muñoz et al. (2010) | QCAZ40040 | JF907493.1 | JX412000.1 |
| *Osornophryne antisana* | Hoogmoed (1987) |  | NA | EU672983.1 |
| *Osornophryne antisana* | Hoogmoed (1987) | DHMECN811 | JF907489.1 | NA |
| *Osornophryne antisana* | Hoogmoed (1987) | DHMECN838 | JF907490.1 | NA |
| *Osornophryne antisana* | Hoogmoed (1987) | QCAZ13320 | JF907452.1 | NA |
| *Osornophryne antisana* | Hoogmoed (1987) | QCAZ40172 | JF907453.1 | JX412007.1 |
| *Osornophryne antisana* | Hoogmoed (1987) | QCAZ40173 | JF907485.1 | JX412008.1 |
| *Osornophryne antisana* | Hoogmoed (1987) | QCAZ40174 | JF907484.1 | JX412009.1 |
| *Osornophryne antisana* | Hoogmoed (1987) | QCAZ46204 | JF907450.1 | JX412025.1 |
| *Osornophryne antisana* | Hoogmoed (1987) | QCAZ46212 | JF907449.1 | JX412026.1 |
| *Osornophryne antisana* | Hoogmoed (1987) | QCAZ48033 | JX418268.1 | JX412029.1 |
| *Osornophryne antisana* | Hoogmoed (1987) | QCAZ48034 | JF907447.1 | JX412030.1 |
| *Osornophryne antisana* | Hoogmoed (1987) | QCAZ48035 | JF907448.1 | JX412031.1 |
| *Osornophryne antisana* | Hoogmoed (1987) | QCAZ48209 | JX418269.1 | JX412032.1 |
| *Osornophryne antisana* | Hoogmoed (1987) | QCAZ48220 | JF907446.1 | JX412033.1 |
| *Osornophryne antisana* | Hoogmoed (1987) | QCAZ48223 | JF907445.1 | JX412034.1 |
| *Osornophryne backshalli sp. nov.* | This paper | DHMECN15260 | PV179439 | PV179459 |
| *Osornophryne backshalli sp. nov.* | This paper | DHMECN15821 | PV179440 | PV179460 |
| *Osornophryne backshalli sp. nov.* | This paper | DHMECN18362 | PV179441 | PV179461 |
| *Osornophryne backshalli sp. nov.* | This paper | DHMECN18363 | PV179442 | PV179462 |
| *Osornophryne backshalli sp. nov.* | This paper | DHMECN18364 | PV179443 | PV179463 |
| *Osornophryne backshalli sp. nov.* | This paper | DHMECN19868 | PV179444 | PV179464 |
| *Osornophryne bufoniformis* | Peracca (1904) | DHMECN6068 | JF907457.1 | JX411980.1 |
| *Osornophryne bufoniformis* | Peracca (1904) | DHMECN1807 | JF907456.1 | NA |
| *Osornophryne bufoniformis* | Peracca (1904) | DHMECN6090 | JF907454.1 | JX411981.1 |
| *Osornophryne bufoniformis* | Peracca (1904) | DHMECN6071 | JF907455.1 | JX411982.1 |
| *Osornophryne bufoniformis* | Peracca (1904) | KU200252 | NA | AF375523.1 |
| *Osornophryne bufoniformis* | Peracca (1904) | QCAZ11472 | AF375497.1 | AF375524.1 |
| *Osornophryne bufoniformis* | Peracca (1904) | QCAZ40003 | JF907430.1 | JX411996.1 |
| *Osornophryne bufoniformis* | Peracca (1904) | QCAZ40121 | JF907432.1 | NA |
| *Osornophryne bufoniformis* | Peracca (1904) | QCAZ40123 | JF907431.1 | JX412003.1 |
| *Osornophryne bufoniformis* | Peracca (1904) | QCAZ45082 | JF907460.1 | JX412021.1 |
| *Osornophryne bufoniformis* | Peracca (1904) | QCAZ45083 | JF907461.1 | JX412022.1 |
| *Osornophryne bufoniformis* | Peracca (1904) | QCAZ45084 | JF907462.1 | JX412023.1 |
| *Osornophryne cf. guacamayo* | Kok et al. 2018 | MF573829.1 | NA | MF573829.1 |
| *Osornophryne cofanorum* | Mueses Cisneros et al. (2010) | DHMECN1579 | JF907439.1 | JX411977.1 |
| *Osornophryne cofanorum* | Mueses Cisneros et al. (2010) | DHMECN1591 | JF907440.1 | JX411978.1 |
| *Osornophryne cofanorum* | Mueses Cisneros et al. (2010) | DHMECN1629 | JF907441.1 | JX411979.1 |
| *Osornophryne guacamayo* | Hoogmoed (1987) | AGG 193 | NA | U52783.1 |
| *Osornophryne guacamayo* | Hoogmoed (1987) | QCAZ4580 | AY326036.1 | AY326036.1 |
| *Osornophryne guacamayo* | Hoogmoed (1987) | QCAZ12240 | JF907469.1 | JX411984.1 |
| *Osornophryne guacamayo* | Hoogmoed (1987) | QCAZ12241 | JF907470.1 | JX411985.1 |
| *Osornophryne guacamayo* | Hoogmoed (1987) | QCAZ17293 | JF907472.1 | JX411988.1 |
| *Osornophryne guacamayo* | Hoogmoed (1987) | QCAZ17294 | JF907473.1 | JX411989.1 |
| *Osornophryne guacamayo* | Hoogmoed (1987) | QCAZ17295 | JF907471.1 | JX411990.1 |
| *Osornophryne guacamayo* | Hoogmoed (1987) | QCAZ2735 | JF907466.1 | JX411991.1 |
| *Osornophryne guacamayo* | Hoogmoed (1987) | QCAZ36882 | JX418267.1 | JX411993.1 |
| *Osornophryne guacamayo* | Hoogmoed (1987) | QCAZ40102 | JF907492.1 | JX412001.1 |
| *Osornophryne guacamayo* | Hoogmoed (1987) | QCAZ40106 | JF907468.1 | JX412002.1 |
| *Osornophryne guacamayo* | Hoogmoed (1987) | QCAZ40138 | JF907463.1 | JX412004.1 |
| *Osornophryne guacamayo* | Hoogmoed (1987) | QCAZ40143 | JF907464.1 | JX412005.1 |
| *Osornophryne guacamayo* | Hoogmoed (1987) | QCAZ40147 | JF907465.1 | JX412006.1 |
| *Osornophryne guacamayo* | Hoogmoed (1987) | QCAZ43370 | JF907474.1 | JX412015.1 |
| *Osornophryne guacamayo* | Hoogmoed (1987) | QCAZ43554 | JF907467.1 | JX412018.1 |
| *Osornophryne guacamayo* | Hoogmoed (1987) | QCAZ4576 | JF907491.1 | JX412024.1 |
| *Osornophryne guacamayo* | Hoogmoed (1987) | QCAZ46661 | NA | JX412027.1 |
| *Osornophryne guacamayo* | Hoogmoed (1987) | QCAZ46662 | JF907475.1 | JX412028.1 |
| *Osornophryne occidentalis* | Cisneros-Heredia and Gluesenkamp (2010) | QCAZ40028 | JF907436.1 | JX411997.1 |
| *Osornophryne occidentalis* | Cisneros-Heredia and Gluesenkamp (2010) | QCAZ43498 | JF907443.1 | JX412017.1 |
| *Osornophryne occidentalis* | Cisneros-Heredia and Gluesenkamp (2010) | QCAZ43652 | JF907444.1 | JX412019.1 |
| *Osornophryne occidentalis* | Cisneros-Heredia and Gluesenkamp (2010) | QCAZ43653 | JF907442.1 | JX412020.1 |
| *Osornophryne percrassa* | Ruiz-Carranza and Hernández-Camacho, 1976 | CMECD146 | NA | JX411976.1 |
| *Osornophryne puruanta* | Gluesenkamp and Guayasamin (2008) | NA | NA | EU672982.1 |
| *Osornophryne puruanta* | Gluesenkamp and Guayasamin (2008) | QCAZ13271 | JF907451.1 | JX411986.1 |
| *Osornophryne puruanta* | Gluesenkamp and Guayasamin (2008) | QCAZ13320 | NA | JX411987.1 |
| *Osornophryne simpsoni* | This paper | DHMECN18365 | PV179445 | PV179465 |
| *Osornophryne simpsoni* | This paper | DHMECN18366 | PV179446 | PV179466 |
| *Osornophryne simpsoni* | This paper | DHMECN18367 | PV179447 | PV179467 |
| *Osornophryne simpsoni* | This paper | DHMECN18368 | PV179448 | PV179468 |
| *Osornophryne simpsoni* | This paper | DHMECN18849 | PV179449 | PV179469 |
| *Osornophryne simpsoni* | This paper | DHMECN18850 | NA | PV179470 |
| *Osornophryne simpsoni* | This paper | DHMECN19866 | PV179450 | PV179471 |
| *Osornophryne simpsoni* | This paper | DHMECN19869 | PV179451 | PV179472 |
| *Osornophryne simpsoni* | This paper | DHMECN15323 | PV179452 | PV179473 |
| *Osornophryne simpsoni* | This paper | DHMECN15325 | PV179453 | PV179474 |
| *Osornophryne simpsoni* | This paper | DHMECN15326 | PV179454 | PV179475 |
| *Osornophryne simpsoni* | This paper | DHMECN18129 | PV179455 | PV179476 |
| *Osornophryne simpsoni* | This paper | DHMECN18130 | PV179456 | PV179477 |
| *Osornophryne simpsoni* | This paper | DHMECN18131 | PV179457 | PV179478 |
| *Osornophryne simpsoni* | This paper | DHMECN18132 | PV179458 | PV179479 |
| *Osornophryne simpsoni* | Páez-Moscoso et al. (2011) | DHMECN5262 | JF907480.1 | JX411983.1 |
| *Osornophryne simpsoni* | Páez-Moscoso et al. (2011) | QCAZ39773 | JF907481.1 | JX411994.1 |
| *Osornophryne simpsoni* | Páez-Moscoso et al. (2011) | QCAZ39774 | JF907478.1 | JX411995.1 |
| *Osornophryne simpsoni* | Páez-Moscoso et al. (2011) | QCAZ39778 | JF907479.1 | NA |
| *Osornophryne simpsoni* | Páez-Moscoso et al. (2011) | QCAZ49776 | JF907476.1 | NA |
| *Osornophryne simpsoni* | Páez-Moscoso et al. (2011) | QCAZ49777 | JF907477.1 | JX412035.1 |
| *Osornophryne simpsoni* | Páez-Moscoso et al. (2011) | QCAZ49778 | NA | JX412036.1 |
| *Osornophryne simpsoni* | Páez-Moscoso et al. (2011) | QCAZ49779 | JF907482.1 | JX412037.1 |
| *Osornophryne simpsoni* | Páez-Moscoso et al. (2011) | QCAZ49781 | JF907483.1 | NA |
| *Osornophryne cf bufoniformis* | Páez-Moscoso et al. (2011) | QCAZ10047 | AF375496.1 | EU672984.1 |
| *Osornophryne cf bufoniformis* | Páez-Moscoso et al. (2011) | NA | NA | EU672981.1 |
| *Osornophryne cf bufoniformis* | Páez-Moscoso et al. (2011) | AGG537 | AF375501.1 | AF375528.1 |
| *Osornophryne cf bufoniformis* | Páez-Moscoso et al. (2011) | QCAZ11471 | AF375499.1 | AF375526.1 |
| *Osornophryne cf bufoniformis* | Páez-Moscoso et al. (2011) | QCAZ9316 | AF375498.1 | AF375525.1 |
| *Osornophryne sumacoensis* | Gluesenkamp (1995) | QCAZ41243 | JF907434.1 | JX412010.1 |
| *Osornophryne sumacoensis* | Gluesenkamp (1995) | QCAZ41246 | JF907437.1 | JX412011.1 |
| *Osornophryne sumacoensis* | Gluesenkamp (1995) | QCAZ41249 | JF907438.1 | JX412012.1 |
| *Osornophryne sumacoensis* | Gluesenkamp (1995) | QCAZ41250 | JF907433.1 | JX412013.1 |
| *Osornophryne sumacoensis* | Gluesenkamp (1995) | QCAZ43379 | JF907435.1 | JX412016.1 |
| *Osornophryne sumacoensis* | Gluesenkamp (1995) | QCAZ4571 | AF375500.1 | AF375527.1 |
