# Supplementary material for: New Andean plump toad of the genus Osornophryne (Anura: Bufonidae) from Cerro Candelaria, Ecuador
Source: PeerJ. 2025 Jul 23;13:e19760. doi: 10.7717/peerj.19760 (PMC12296580; doi:10.7717/peerj.19760)
Supplement: Supplemental Information 2 [file peerj-13-19760-s002.docx]

**Examined specimens**

*Osornophryne sumacoensis* (3): Ecuador: Napo (1): from at 2500 m on slope of crater 5 km east of summit of Volcan Sumaco (-0.59122, -77.6210; 2055 m) QCAZ 4570; Ecuador: Orellana: (2) Loreto (-0.5684, -77.5956; 2497 m) MZUTI 6412, (-0.59122, -77.58767; 1901 m) MZUTI 6912. *Osornophryne simpsoni* (7): Ecuador: Pastaza (1): (-1.2762, -78.07254; 2263 m) QCAZ 49774; Ecuador: Napo (6): (-0.93765, -77.94848; 2196 m) DHMECN 15324, (-0.93402, -77.95841; 2539 m) DHMECN 15338, (-1.436789, -78.301019; 2565 m) DHMECN 18365, (-1.43841, -78.301946; 2626 m) DHMECN 18370, (-1,433301, -78,2613; 2405 m) DHMECN 18128 and DHMECN 18132. *Osornophryne occidentalis* (6)*:* Ecuador: Carchi (5): Espejo (0,82359, -78,09471; 2512 m) DHMECN 18998, (0,81943, -78,09339; 2549 m) DHMECN 19000, (0,82493, -78,0962; 2456 m) DHMECN 19001; Tulcan (0,86642, -78,20303; 2259 m) DHMECN 19404, (0.83239, -78.24377; 2390 m) DHMECN 19565; Ecuador: Imbabura (1): Intac (0.34003, -78.43871; 2700 m) DHMECN 20004.
